# A Current Overview of the Biological Effects of Combined Space Environmental Factors in Mammals

**DOI:** 10.3389/fcell.2022.861006

**Published:** 2022-04-12

**Authors:** Ying Xu, Weiwei Pei, Wentao Hu

**Affiliations:** ^1^ State Key Laboratory of Radiation Medicine and Protection, Soochow University, Suzhou, China; ^2^ School of Radiation Medicine and Protection, Suzhou Medical College of Soochow University, Suzhou, China; ^3^ Collaborative Innovation Center of Radiological Medicine of Jiangsu Higher Education Institutions, Suzhou, China

**Keywords:** microgravity, space radiation, hypomagnetic field, space exploration, combined biological effect

## Abstract

Distinct from Earth’s environment, space environmental factors mainly include space radiation, microgravity, hypomagnetic field, and disrupted light/dark cycles that cause physiological changes in astronauts. Numerous studies have demonstrated that space environmental factors can lead to muscle atrophy, bone loss, carcinogenesis, immune disorders, vascular function and cognitive impairment. Most current ground-based studies focused on single environmental factor biological effects. To promote manned space exploration, a better understanding of the biological effects of the spaceflight environment is necessary. This paper summarizes the latest research progress of the combined biological effects of double or multiple space environmental factors on mammalian cells, and discusses their possible molecular mechanisms, with the hope of providing a scientific theoretical basis to develop appropriate countermeasures for astronauts.

## Introduction

So far, more than 500 astronauts have been sent into space to conduct space exploration missions, who are inevitably faced with a harsh space environment when conducting research activities. Space environmental factors include microgravity, hypomagnetic field, circadian rhythm different from the earth’s environment, space ultraviolet, ionizing radiation, high vacuum, extreme temperature and noise (Thirsk et al., 2009; Horneck et al., 2010). After more than 50 years of space explorations, a large number of studies have proved that the space environment has adverse effects on organisms. Astronauts carrying out space exploration missions may present changes in gene regulation and genomic integrity, as well as molecular and cellular biological changes such as mitochondrial dysfunction. These changes will increase health risks, including muscle atrophy and bone loss, carcinogenesis, immune disorders, changes in vascular function, and cognitive impairment (Williams et al., 2009; Garrett-Bakelman et al., 2019).

At present, studies on single factor biological effects of the space environment mainly focus on space radiation, microgravity, hypomagnetic field and circadian rhythm. There are three main sources of space ionizing radiation: galactic cosmic rays (GCR) containing high-energy protons and heavy ions, solar particle events (SPE) containing medium and high-energy protons, and trapped radiation belt (TRB) dominated by 10 MeV protons and electrons (Simonsen et al., 2020). International Space Station (ISS) orbits the Earth at an altitude of about 350 km, with radiation levels of 0.4–1.0 mSv/days, which is about 100 times the level of radiation on Earth’s surface; for missions beyond low Earth orbit, such as lunar and Mars exploration, astronauts will be more affected by GCR (Yatagai and Ishioka, 2014). In addition, primary space radiation from the universe reacts with the spacecraft bulkhead to generate secondary rays such as neutrons, X-rays and γ-rays, which can produce reactive oxygen species (ROS) more effectively than primary space radiation (Inozemtsev et al., 2018). The main effects of space radiation on organisms are genotoxic effects (such as single-strand and double-strand DNA breaks, chromatin structure destruction, base tautomerism), and other effects including oxidative stress, immune disorders and central nervous system damage (Yatagai and Ishioka, 2014; Kokhan et al., 2019a). Microgravity is another important space environmental factor and is about 10^−4^–10^−6^ g in the interplanetary space, which is a state of physical gravity reduction. The change of gravity will affect cell proliferation, differentiation, signal transduction, gene expression and membrane rearrangement (Wuest et al., 2018; Yatagai et al., 2019), resulting in physiological changes at the tissue or organ level during space flight. The effects of microgravity on organisms mainly include changes in gene expression, chromosome aberration, apoptosis, immunosuppression, cardiovascular disease, skeletal muscle atrophy and bone loss (Girardi et al., 2012; Lin et al., 2020; Neelam et al., 2020). The commonly used equipment and models for ground simulation of microgravity include rotating wall vessels (RWV), 2D and 3D clinostat, random positioning machine (RPM), hindlimb unloading (HLU) model, anti-magnetic levitation model and parabolic flying aircraft (Herranz et al., 2012; Herranz et al., 2013; Ghosh et al., 2016; Stervbo et al., 2018; Paul et al., 2021). For humans, the head-down tilt bed rest model is usually used to study the effect of microgravity on bone, muscle, cardiovascular system (Liang et al., 2014; Moreno-Villanueva et al., 2017). There is limited research on the biological effects of the space environments’ hypomagnetic field combined with light/dark cycle dysregulations. The strength of the geomagnetic field (GMF) is about 50 μT, while the intensity of the magnetic field in outer space is very low, called hypomagnetic field (HyMF/HMF, |B| < 5 μT) (Mo et al., 2014; Yang et al., 2018). Mars magnetic field (< 5 μT), lunar magnetic field (< 300 nT) and interstellar space magnetic field (several nT) all belong to hypomagnetic field. Astronauts are inevitably exposed to HMF conditions when performing long-cycle and long-distance space missions. HMF can interfere with a variety of functions of organisms, including gene expression, individual growth, embryonic development, learning and working abilities (Zhang et al., 2021a; Zhang et al., 2021b). The orbit of a spacecraft in low orbit around the earth is about 90 min (Dijk et al., 2001; Thirsk et al., 2009). It means that 16 days and night cycles in 24 h. Such a light-dark cycle pattern is inconsistent with the rhythm of human life on the earth. This seriously affects the biological clock of astronauts, resulting in a decline in sleep quality, appetite, learning and memory capacity (Taillard et al., 2021; von Gall, 2022). During missions to Mars, the light-dark cycle is variable rather than fixed outside the spacecraft and astronauts will be exposed to artificial light rather than the natural light on earth for a long time in the spacecraft. High-fidelity ground simulation studies of Mars missions showed that light exposure patterns during missions to Mars led to melatonin cycle disorder, autonomic nervous system parasympathetic dysfunction, vigilance deficits, shorter total sleep time and lower sleep efficiency (Basner et al., 2013; Vigo et al., 2013; Gemignani et al., 2014). Considering the limited experimental opportunities on spacecrafts or space stations, a number of ground-based studies have been carried out on the effects of single space environmental factor on organisms in order to protect the life and health of astronauts, but the research on the combined biological effects of double or multiple environmental factors is relatively limited. In this paper, the latest research will be reviewed.

## Combined Biological Effects of Space Environmental Factors in Mammals

### Space Radiation and Microgravity

Research of the biological effects of space environmental factors usually takes space radiation and microgravity as characteristic factors. Many studies have discussed the combined effects of space radiation and microgravity in mammals of the two factors to explain how space environment can cause the physiological changes in astronauts, including the effects on bones, nervous system, circulatory system and reproductive system, as demonstrated in Table 1. The negative effects of microgravity on bone are obvious, and most of the studies about microgravity and radiation also focus on their combined effect on bone formation and functions. It was found that high LET iron ion radiation (0.5 or 2 Gy) aggravated the decrease of bone strength caused by lumbar decommissioning in a HLU model of 4-month-old male C57BL/6J mice, and partially prevented the recovery of cancellous bone microstructure caused by bone unloading, resulting in the loss of bone integrity (Alwood et al., 2010). Proton irradiation (1 Gy) combined with HLU was also reported to reduce the bone strength and mechanical properties of the femur and tibia in 15-week-old female C57BL/6 mice (Lloyd et al., 2012). Low dose X-rays (25 mGy) combined with HLU could reduce the trabecular mass of 10-week-old male C57BL/6J mice, but there was no significant difference compared with the experimental group treated with HLU alone, while the decrease of bone surface area and femoral cortical thickness only appeared in the HLU combined radiation group, and it was found that the loss of cortical bone and the decrease of trabecular mass were caused by the absorption of osteoclasts, which are multinucleate giant cells formed by the fusion of mononuclear macrophages (Farley et al., 2020). The effect of high LET radiation combined with partial weightlessness on bones was also studied. Female BALB/cByJ 4-month-old mice were exposed to low dose and high LET silicon ion radiation (0.17 Gy single radiation, 0.5 Gy single radiation and fractionated radiation) under partial weightlessness (G/6 to simulate lunar gravity). It was noted that the combination of the two factors had a negative effect on the maintenance of bone mass by both reducing bone formation and increasing bone resorption, and the impaired bone formation response was related to the inhibited Wnt signaling (Macias et al., 2016), the increased Runx2 and decreased Caspase-3 mRNA expression (Dong et al., 2021). On the whole, most studies showed that microgravity-induced bone loss and impaired bone mechanical properties were aggravated by ionizing radiation exposure.

**TABLE 1:** Studies reporting the combined effects of irradiation and microgravity in mammals.

Studied materials	Experimental treatments	Combined biological effects	Refs
16-week-old male C57BL/6 mice	HLU (3 days) + iron ions (1 Gy) + HLU (10–13 days)	Impairment of vasodilator function in resistance arteries	Ghosh et al. (2016)
4-month-old male C57BL/6J mice	HLU (11 days) + iron ions (0.5 Gy) + HLU (3 days)	Decreased of bone strength and loss of bone integrity	Alwood et al. (2010)
15-week-old female C57BL/6 mice	protons (1 Gy) + HLU (4 weeks)	Decrease of trabecular bone volume fraction, connectivity density, and trabecular number	Lloyd et al. (2012)
10-week-old male C57BL/6J mice	HLU (7 days) + X-rays (25 mGy) + HLU (7 days)	Decrease of the trabecular mass, bone surface area and femoral cortical thickness	Farley et al. (2020)
Female BALB/cByJ 4-month-old mice	silicon ions (0.5 Gy) + G/6 (21 days), silicon ions (0.17 Gy) + G/6 (3 days) + silicon ions (0.17 Gy) + G/6 (5 days) + silicon ions (0.17 Gy) + G/6 (13 days)	Decrease of bone formation while increase of bone resorption	Macias et al. (2016)
7-week-old male SD rats	X-rays (2 Gy) + HLU (4 weeks)	Increased bone loss and Caspase-3 mRNA expressions and decreased Runx2 mRNA expressions	Dong et al. (2021)
6-month-old female C57BL/6J mice	γ-rays (0.04 Gy, 0.01 cGy/h)-HLU (21 days)	Late onset neurological sequelae	Overbey et al. (2019)
Weighing 170–190 g male Wistar rats	HLU (8 days) + γ-rays (4.5 Gy) + HLU (13 days)	HPA axis and immune dysfunction	Zhu et al. (2020)
6-week-old male Wistar rats	HLU (7 days) + γ-rays (3 Gy for whole-body) + HLU (7 days) + protons (1.5 Gy for head)	Antagonistic effects on the psycho-emotional status and cognitive abilities	Kokhan et al. (2017)
3-months -old male Wistar rat	6*[γ-rays (0.5 Gy for whole-body) + HLU (5 days)] + protons (1.5 Gy for head)	Recovery of impaired motor, autonomic, exploratory behavior and long-term contextual memory	Kokhan et al. (2019b)
6-month-old female C57BL/6J mice	γ-rays (0.04 Gy, 0.01 cGy/h)-HLU (21 days)	Decreased level of superoxide dismutase	Mao et al. (2016)
6-month-old female C57BL/6J mice with Nox2-KO	γ-rays (0.04 Gy, 0.01 cGy/h)-HLU (21 days)	Inhibition of oxidative response	Mao et al. (2017)
6-month-old and female C57BL/6J mice	γ-rays (0.04 Gy, 0.01 cGy/h)-HLU (21 days)	Persistent inflammation and anemia	Paul et al. (2021)
8- to 12-week-old C57BL/6 male mice	γ-rays (0.5 Gy)-HLU (a month)	Changes in proportions of immune cells in the thymus	Sadhukhan et al. (2021)
6- to 8-week-old female ICR mice	HLU (3 days) + protons (2 Gy)	Reduced number of spleen T lymphocytes and toxic T cells	Sanzari et al. (2013)
6-month-old male C57BL/6J mice	HLU (7 days) + protons (50 cGy) + HLU (7 days)	Decreased B cell count in retinal vessels, increased NK cell count	Mao et al. (2019)
16-week-old male C57BL/6J mice	HLU (3 days) + γ-rays (200 cGy) + HLU (12 days)	Obstruction of skeletal muscle artery vasodilation	Prisby et al. (2016)
6-month-old female C57BL/6J mice	γ-rays (0.04 Gy, 0.01 cGy/h)-HLU (21 days)	Decreased GSH:GSSG ratio, SAM:SAH ratio and inflammation marker CD-2	Seawright et al. (2017)
10-week-old male Swiss Webster mice	HLU (7 days) + carbon ions (0.25, 0.5, 1, 2 Gy)	Increased spermatogenic cell apoptosis and DNA damage, decreased sperm count and survival rate	Li et al. (2013)
Weighing 30–35 g male Kunming mice	HLU (7 days) + carbon ions (0.2, 0.4, 0.8, 1 Gy)	Decreased number of spermatozoa, primary spermatocytes and spermatogonia, increased apoptosis	Liu et al. (2013)

“+”: sequential treatments; “-”: simultaneous treatments.

The combined effect of microgravity and radiation also has an effect on the nervous system, usually in an antagonistic manner. In the striatum, the content of 5-hydroxytryptamine (5-HT) metabolite 5-hydroxyindoleacetic acid (5-HIAA) increased under the action of HLU simulated microgravity alone, and γ-ray irradiation (3 Gy) combined with microgravity could normalize the content of 5-HIAA (Shtemberg et al., 2014). In the prefrontal cortex (PFC) and amygdala of Long-Evans rats, the combined effects of radiation (whole body γ-irradiation of 3 Gy and carbon-ion head irradiation of 1.5 Gy) and microgravity (12 days) led to their antagonistic effects on 5-HT neurotransmitters. Moreover, irradiation was dominant to reduce 5-HT metabolism (Kokhan et al., 2019a). Neural readaptation occurred in 6-month-old female C57BL/6J mice treated with low dose and low dose rate γ-rays (0.04 Gy, 0.01 cGy/h) and HLU for 21 days, which is a dynamic process involving pathways regulating neuronal function and structure and leads to delayed neurological sequelae (Overbey et al., 2019). γ-ray irradiation (4.5 Gy) can antagonize the stress response of the hypothalamus-pituitary-adrenocortical (HPA) axis and macrophages induced by microgravity in male Wistar rats, and aggravate T cell dysfunction, resulting in HPA axis and immune dysfunction (Zhu et al., 2020). Simulated microgravity and ionizing radiation (γ-ray whole-body exposure of 3 Gy and proton head exposure of 1.5 Gy) also antagonized the psychological and emotional state and cognitive ability of 6-week-old male Wistar rats, which was found to be related to the changes of monoamine content in PFC, hippocampus and hypothalamus (Kokhan et al., 2017). Further research showed that combined microgravity and radiation affected the conversion rates of 5-HT and dopamine (DA) in PFC, hippocampus and striatum of 3-month-old male Wistar rats. This reduced the adverse effects induced by microgravity and radiation alone, such as the recovery of the impaired motor activity and long-term contextual memory, and the suppressed orientation and exploratory behavior. It is speculated that 5-HT receptor 5-HT2a in PFC and DA receptor D2 in hippocampus are involved (Kokhan et al., 2019b). In brains of 6-month-old female C57BL/6J mice treated with HLU model (21 days) combined with low dose rate radiation (122 keV/μm, γ-rays of 40 mGy), it was found that the level of specific oxidation marker 4-HNE protein in nerve increased and the level of superoxide dismutase (SOD) decreased. It is estimated that the increase of neuronal damage caused by long-term exposure to simulated microgravity and low dose rate radiation may be related to the increase of oxidative pressure and the decrease of antioxidant defense ability (Mao et al., 2016). In addition, it was also discovered that oxidative stress in brains of 6-month-old female B6.129S6-CYBBM mice knocked out of NADPH oxidase Nox2 gene was inhibited by 0.04 Gy γ-rays and HLU simulated microgravity. It is speculated that Nox2 may be involved in space environment-induced oxidative stress (Mao et al., 2017).

Circulatory and immune systems were also found to be affected by the combined effect of microgravity and radiation. 6-month-old female C57BL/6J mice after continuous low-dose γ-irradiation (a total dose of 0.04 Gy) and HLU (21 days) treatment showed persistent low-grade inflammation and decreased red blood cell quality, which is suggestive of anemia (Paul et al., 2021). When 8- to 12-week-old male C57BL/6 mice were simultaneously exposed to chronic γ-radiation (a total dose of 0.5 Gy) and HLU simulated microgravity for a month, the proportion of immune cells in their thymus altered, but not in the spleen (Sadhukhan et al., 2021). Compared with the action of HLU alone, proton radiation of 2 Gy combined with HLU could significantly reduce the number of spleen T lymphocytes and cytotoxic T lymphocytes in 6–8-week-old female ICR mice, and the activation of T lymphocytes was inhibited and their proliferation was significantly decreased (Sanzari et al., 2013). The level of endothelial nitric oxide synthase (eNOS) in the retina of 6-month-old male C57BL/6J mice increased significantly after 50 cGy proton radiation combined with HLU treatment, the B cell count in retinal vessels decreased significantly, and the NK cell count increased (Mao et al., 2019). Compared with HLU (15 days) and γ-irradiation of 200 cGy (84 cGy/min) alone, the combined effect of them did not further reduce the endothelium-dependent vasodilation function of gastrocnemius artery in 16-week-old C57BL/6J male mice, but increased the mRNA expression of eNOS and decreased the vasodilation mediated by endothelium-derived relaxing factor NO, suggesting that the NOS signal pathway may be uncoupled. The mechanism of vasodilation has shifted from a mechanism that mainly depends on NO to a mechanism that may be more dependent on PGI2 signals. It is also concluded that the damage of endothelium-dependent vasodilation mediated by microgravity and radiation is closely related to the changes of trabecular volume and microstructure. It speculates that NO couples vascular signals to bone remodeling (Prisby et al., 2016). There are also studies on the effects of high LET radiation combined with microgravity on vasodilation. The researchers treated 16-week-old male C57BL/6 mice with iron ion beam irradiation of 1 Gy (10 cGy/min) and HLU (13–16 days). It was found that the combined effect could further damage the endothelium-dependent vasodilation function of gastrocnemius artery through NOS signal pathway, and had a negative effect on eNOS, xanthine oxidase XO and SOD-1 protein levels. In addition, it was also found that the decrease of endothelium-dependent vasodilation was associated with lower cancellous bone volume fraction (Ghosh et al., 2016). 21 days after 6-month-old female C57BL/6J mice were exposed to HLU and γ-rays (0.01 cGy/h, 0.04 Gy), GSH:GSSG ratio, SAM:SAH ratio and inflammation marker CD-2 protein content decreased. These results suggest that exposure to low dose rate and low dose radiation combined with microgravity can reduce the potential of DNA methylation in heart tissue (Seawright et al., 2017).

Similar findings were observed in another study about the combined effect of microgravity and radiation on the reproductive system. HLU combined with 0.25–2 Gy carbon-ion radiation was utilized to treat 10-week-old male Swiss Webster mice. It was found that the combined action of HLU and carbon ion radiation promoted the expression of p53, Bax and PCNA in spermatogenic cells and increased cell apoptosis, while reduced sperm count and survival rate and aggravated sperm DNA damage in mice. It is speculated that one of the reasons for the decline of male fertility in space environment is sperm DNA damage (Li et al., 2013). Compared with normal gravity, the number of spermatozoa, primary spermatocytes and spermatogonia of male Kunming mice reduced significantly under microgravity simulated by HLU and carbon ion irradiation (0.4–1 Gy). Radiation at the dose of 0.8 Gy or above combined with microgravity can significantly up-regulate the ratio of Bax/Bcl-xL. It is assumed that the aggravation of germ cell injury is caused by the increase of apoptosis caused by mitochondrial apoptosis pathway (Liu et al., 2013). These results suggest that the combined effects of microgravity and radiation have obvious adverse effects on the male reproductive system, usually in a synergistic manner, while there are few studies on the female reproductive system.

The research of microgravity combined radiation is increasing gradually, and some effects as well as the underlying mechanisms were demonstrated. However, the research of the combined effect is still in the preliminary stage, and the mechanism underlying most phenomena is still lacking. There are also shortcomings in the field. Space radiation environment is dominated by GCR particles at a relatively low dose rate (about 1.84 mSv/day) during manned interplanetary flights (Zeitlin et al., 2013). However, most of radiation types used in current research are photons delivered as acute irradiation, while there are few studies employing protons and heavy ions that are delivered as chronic irradiation. Many studies have shown that acute and chronic exposures cause different biological effects. For example, acute and chronic radiation exposure have dissimilar effects on CNS, which in turn affects the behavioral and cognitive performance on mice (Holden et al., 2021), as well as the phenotype of immune cells and genomic response (Kovalchuk et al., 2004; Chaudhry et al., 2012; Sadhukhan et al., 2021). Therefore, the dose rate of radiation should be taken into account in the study of the combined effects of radiation. Due to analog limitations of studying spaceflight environmental factors on Earth, space radiation co-exists with the microgravity during space exploration mission duration. Current ground-based studies mostly treat the samples by ionizing radiation and simulated microgravity in a sequential manner, which is quite different from the real space environment and the yielded results are limited in explaining the biological effects observed in space. Thus, further research into high fidelity modeling schemes for spaceflight is still required.

### Hypomagnetic Field and Microgravity

The magnetic and gravitational fields are a natural part of a habitable environment on Earth, and both inevitably change in space. Numerous studies have shown that microgravity can cause bone loss and muscle atrophy, yet, the combined effect between hypomagnetic field and microgravity are limited (Table 2). Studies have shown that HMF (< 300 nT) can aggravate bone loss and change the biomechanical properties of the femur in HLU male Sprague-Dawley (SD) rats. At the same time, an obvious expression of RANKL (Receptor Activator of Nuclear Factor Kappa B Ligand) and increase of serum iron concentration were observed in the trabeculae of the femur. Elevated expression of RANKL is the main cause of bone loss (Jia et al., 2014). It was also found in HLU 8-week-old male C57BL/6 mice that HMF (<300 nT) promoted bone loss of femur and had a negative effect on the biomechanical properties of the femur, and the accumulation of iron in serum, liver, spleen and bone of mice was significantly higher than that of microgravity group and HMF group (Yang et al., 2018). It is speculated that iron overload causes oxidative stress and promotes bone loss. Compared with HLU combined with GMF and reloading for a period of time, the bone mineral content of femur and tibia of 6-week-old male C57BL/6 mice reloaded with HLU combined with HMF (B < 300 nT) was lower, the microstructure and mechanical properties of femur were worse, the unbalanced bone remodeling of the tibia was less ideal, and the content of iron in serum, tibia, liver and spleen was higher. The iron content in bone, liver and spleen of mice treated with iron chelated agent deferoxamine (DFO) decreased. DFO can significantly reduce the bone loss induced by microgravity in the HMF reload group, implying that HMF inhibits the recovery of bone loss induced by microgravity. It may be due to the increase of ferritin Hepcidin expression and the decrease of membrane transporter FPN expression, which causes iron accumulation to return to physiological level (Xue et al., 2020b).

**TABLE 2:** Studies reporting combined biological effects of hypomagnetic field and microgravity in mammals.

Studied materials	Experimental treatments	Combined biological effects	Refs
8-week-old male C57BL/6 mice	HMF (<300 nT)-HLU (4 weeks)	Accumulation of iron, bone loss and negative effect on the biomechanical properties of the femur	Yang et al. (2018)
Weighing 260 ± 10 g male SD rats	HMF (<300 nT)-HLU (4 weeks)	Expression of RANKL and serum iron concentration increase	Jia et al. (2014)
6-week-old male C57BL/6 mice	HLU (4 weeks) + HMF (<300 nT, 4 weeks)	Lower bone mineral content, iron accumulation, unbalanced bone remodeling	Xue et al. (2020b)
Weighing 260 ± 10 g male SD rats	HMF (<300 nT)-HLU (4 weeks)	The dielectric properties of the gastrocnemius muscle are smaller than that of both alone	Ding et al. (2014b)
Human bronchial epithelial cells BEAS-2B	HMF (<50 nT, 1 week) + X-rays (2, 4, 6 Gy)	Cell survival increase, less DNA damage and more efficient damage repair	Xue et al. (2020a)

“+”: sequential treatments; “-”: simultaneous treatments.

In addition, a small amount of the research is dedicated to the combined effect of microgravity and hypomagnetic field on the dielectric properties of tissue, as demonstrated in Table 2. There was no significant change in the dielectric properties of the gastrocnemius muscle of male SD rats treated with HLU for 4 weeks, but the electrical conductivity of gastrocnemius muscle was increased when the intensity of HMF was lower than that of 300 nT alone. At the same time, it is found that the combined effect of HLU and HMF on the dielectric properties of the gastrocnemius muscle is smaller than that of both alone, rather than a simple additive relationship (Ding et al., 2014b). HLU and HMF alone or in combination have different effects on the dielectric properties of the spleen, whole blood and testis of male SD rats, and the degree of change varies with different tissues (Ding et al., 2014a). These experiments suggest that the dielectric properties of biological tissues in the space environment are different from those in normal conditions, which are worthy of further study, so as to expand the application of biological physiology and pathology detection and monitoring in the space environment.

The above experiments suggest that HMF can exacerbate microgravity-induced bone loss and this process may be caused by iron accumulation. In addition, HMF combined with microgravity can affect the dielectric properties of tissues and organs. These studies provide a basis for the role of GMF and HMF in the recovery of bone loss and muscle atrophy induced by microgravity. Beyond these, there is little research on other systems. HMF can affect the function of multiple systems in an organism, especially in the central nervous system (Zhang et al., 2021b). Similarly, microgravity can affect the central nervous system while the combined effects of HMF and microgravity is yet to be elucidated.

### Multiple Space Environmental Factors

To simulate the space environment as accurately as possible, some researchers have carried out studies on the combined biological effects greater than two environmental factors in mammals. They used circadian rhythm changes (45 min bright: 45 min dark) combined with HLU and 4 Gy X-ray irradiation to study the combined effects on the hindlimb bones of rats. It was found that the combination of the three factors could significantly reduce bone biomechanical properties, bone mineral density and bone trabecular parameters, resulting in bone loss. However, there was no significant difference between the HLU group and the combined three-factor group. The results showed that the change of circadian rhythm had no significant effect on bone parameters (Zhang et al., 2018). Other researchers placed SD rats in a new type of cage that could simulate circadian rhythm disorder (12 h bright: 12 h dark), microgravity (HLU model), noise (65 dB) and claustrophobic environment. It was found that the rats had depressive behavior, with upregulated expression of splice-related proteins, but downregulated expression of oxidative phosphorylation, glutamatergic and GABA-ergic synaptic related proteins and post-synaptic density protein 95 (PSD-95) proteins (Min et al., 2021). It is speculated that these differentially expressed proteins may be markers of mental disorders. However, the health effects caused by the combination of circadian rhythm changes and other space environmental factors have not been reported.

## Combined Biological Effects of Space Environmental Factors in Human Cells

### Space Radiation and Microgravity

According to the classic Target Theory, nuclear DNA damage induced by space radiation plays a key role in the cell fate determination in cells exposed to space radiation. Whether microgravity affects radiation-induced DNA damage and subsequent cell fate depends on the cell type and growth condition (Lu et al., 2017). For example, the interaction between these two environmental factors inducing chromosome aberration (CA) has been controversial. Microgravity simulated by RWV had no effect on the CA of human lymphocytes induced by X-rays or proton beams (0–6 Gy) (Manti et al., 2005). However, the CA of human fibroblasts or human lymphoblastic TK6 cells exposed to radiation (1.5 Gy X-rays or 0.5 Gy carbon-ion beams) and clinostat simulated microgravity (24 h) increased (Hada et al., 2019; Yamanouchi et al., 2020), and the CA of human lymphocytes exposed to X-rays (1.5 Gy) and clinostat simulated microgravity also increased (Mosesso et al., 2001). In addition, the combined effect of microgravity and space radiation on DNA damage repair is also controversial. Under simulated microgravity, the efficiency of DNA damage repair of human lymphocytes exposed to 5 Gy γ-rays decreased (Mognato et al., 2009). However, when human fibroblasts irradiated by 5 Gy or 10 Gy X-rays were carried into space by the space shuttle Columbia carrying out the STS-65 mission, there is no significant difference between the DNA damage repair efficiency of cells under real space microgravity and ground gravity (Horneck et al., 1996). In another study employing human lymphoblastoid TK6 cells, it was found that microgravity simulated by RWV reduced the level of γ-ray irradiation-induced apoptosis while enhanced genomic damage as manifested by HPRT mutant frequency and the micronucleus formation (Canova et al., 2005). However, Dang et al. found that microgravity simulated by RWV aggravated carbon ion radiation-induced cell apoptosis mediated by a ROS-sensitive signaling pathway in human B lymphoblast HMy2.CIR cells (Dang et al., 2014). As demonstrated in Table 3, it can be concluded that microgravity may present synergistic, additive or antagonistic effect on DNA and cellular damage induced by ionizing radiation.

**TABLE 3:** Studies reporting the combined effects of space environment in human cells.

Studied materials	Experimental treatments	Combined biological effects	Refs
Human peripheral blood lymphocytes	γ-rays (0.2, 2 Gy) + RWV (4, 24 h)	Decreased number of radio-responsive miRNAs	Girardi et al. (2012)
Human lymphocytes	X-rays/protons (1–6 Gy) + RWV (24 h)	No effect on CA	Manti et al. (2005)
Human fibroblast 1BR-hTERT cells	X-rays/carbon ions (0.5, 1.5 Gy)-3D clinostat (24 h)	Increased CA	Hada et al. (2019)
Human lymphoblastic TK6 cells	X-rays/carbon ions (0.5, 1.5 Gy)-3D clinostat (24 h)	Increased CA	Yamanouchi et al. (2020)
Human lymphocytes	X-rays (1.5 Gy) + cuvette clinostat (2 h)	Increased CA	Mosesso et al. (2001)
Human peripheral blood lymphocytes	γ-rays (5 Gy) + RWV (0.5, 2, 6, 24 h)	Decreased DNA damage repair	Mognato et al. (2009)
Human skin fibroblast NHF-23 cells	X-rays (5, 10 Gy) + real microgravity (4.5 h)	No effect on DNA damage repair	Horneck et al. (1996)
Human lymphoblastoid TK6 cells	γ-rays (1, 2, 4 Gy) + RWV (24 h)	Decreased apoptosis	Canova et al. (2005)
Human B lymphoblastoid HMy2.CIR cells	RWV (30 min) + carbon ions (0.2, 0.4, 0.6, 0.8 Gy)	Increased cell survival and apoptosis	Dang et al. (2014)
Human peripheral blood lymphocytes	X-rays/γ-rays (1, 2 Gy) + RWV (24 h)	Increased HPRT mutation frequency	Mognato and Celotti (2005)
Human fibroblasts 1BR-hTERT cells	X-rays/carbon ions (1 Gy, 0.03 Gy/min)-3D clinostat (48 h)	DNA damage and failure of cell cycle checkpoint block	Ikeda et al. (2019)
Human lymphoblast TK6 cells	γ-rays (2 Gy) + RWV (24 h)	Additive/synergistic effects on RNA expression patterns/levels	Fu et al. (2020)
Human lung epithelial cells BEAS-2B	3D clinostat (48 h) + X-rays (2 Gy)	Increased DNA damage, apoptosis and RAC2 expression and inhibition of proliferation and survival additively	Tan et al. (2020)

“+”: sequential treatments; “-”: simultaneous treatments.

Several studies have also tried to clarify the underlying molecular mechanisms of cellular damage caused by microgravity combined with radiation. Human peripheral blood lymphocytes (PBL) were incubated in microgravity simulated by RWV after exposure to X-rays or γ-rays (1 or 2 Gy). Compared with irradiated PBL incubated in normal gravity, the mutation frequency of HPRT in microgravity increased, but the transcription of DNA repair-related genes did not change significantly, indicating that the increased mutation frequency had nothing to do with transcriptional impairment. It may be related to the decreased activity of DNA damage sensing proteins (Mognato and Celotti, 2005). Ikeda et al. (2019) maintained human fibroblasts under 3D clinostat simulated microgravity and exposed them to synchronized 1 Gy carbon-ion radiation. It was found the expression of cell cycle inhibitory genes decreased, while the expression of cell cycle promoting genes increased, which may lead to failure of cell cycle checkpoint block and increased genomic instability. By analyzing the mRNA and miRNA expression profiles of human PBL treated by γ-ray irradiation (0.2 or 2 Gy) and microgravity stimulated by RWV for 4 h or 24 h, it was found that microgravity reduced the number of radiation-responsive miRNAs and changed the gene expression of DNA damage repair pathway, suggesting that simulated microgravity can affect the radiation-induced DNA damage response (Girardi et al., 2012). RWV simulated microgravity and 2 Gy γ-ray irradiation were shown to additively change the expression patterns of RNAs while synergistically altering their expression levels in human lymphoblast TK6 cells, among which miRNAs and lncRNAs were predicted to regulate immune/inflammatory response through their target genes (Fu et al., 2020). Tan et al. (2020) suggested that 3D clinostat simulated microgravity for 48 h and 2 Gy X-rays could additively inhibit the proliferation and induce the apoptotic and DNA double-strand breaks of human lung epithelial BEAS-2B cells, possibly due to the upregulation of RAC2

Considering the above-mentioned studies, the effects of radiation quality, types of samples, biological endpoints and even sample collection time points all show an impact on the research results, which should be carefully considered in the follow-up studies. Though various progresses have been achieved, the specific molecular targets mediating combined biological effects of microgravity and radiation remain to be clarified. In addition, the studies on the combined effect of space radiation and microgravity on cells are mainly focused on genetic material, and there is a lack of studies on phenotypic microstructure or submicron structure effects.

### Space Radiation and Hypomagnetic Field

In the deep space environment, astronauts will be more susceptible to space radiation-induced adverse effects after leaving the protection of the earth’s magnetic field, which highlights the importance of the combined biological effect study of hypomagnetic field and space radiation. By studying DNA damage and repair of immortalized human bronchial epithelial BEAS-2B cells exposed to HMF (<50 nT) after 2 Gy X-ray irradiation, Xue et al. (2020) found increased survival, less DNA damage and more efficient DNA damage repair, suggesting potential application of HMF in radiation protection. This was also the first that identified that HMF participates in the process of DNA damage repair induced by irradiation.

## Future Challenges

Human exploration of space is rapidly developing, which requires deeper studies and understanding of space medicine and space biology. This paper summarizes the research progress on the combined biological effects of space environmental factors and the underlying mechanism in mammalian cells and provides suggestions for the further study of the harmful physiological effects of space environment.

Throughout this review, current research on the combined biological effects of space environmental factors needs to be improved in the following aspects: 1) Except for the study of microgravity combined with space radiation, there are very few studies on the combined effects of other space environmental factors. 2) It is still uncertain which specific genes or signaling pathways mediate the coupling of the biological effects of the different space environmental factors, leading to hesitation in moving forward in both diagnosis and targeted treatment of related diseases. 3) It is speculated that radiation-induced non-targeted effect may play a significant role in the biological outcomes induced by low-dose and low-dose rate space radiation (Shuryak et al., 2021). Therefore, influence of other space environmental factors on the radiation-induced non-targeted effect remains to be clarified. 4) No full-scale ground analogs that can completely model the spaceflight environment (LEO or beyond LEO), including microgravity, hypomagnetic field, disrupted light/dark cycles, space radiation, and noise, while the biological experimental opportunities basing on spacecrafts or space stations are very limited.

In conclusion, there is an urgent need to build a ground-based research platform for synchronized spaceflight factors space environment simulation, in order to carry out biological experiments at the cell, tissue and individual levels, and to enrich the knowledge of the combined biological effects of space environmental factors on Earth as well as their interactive mechanisms, in the hope of providing a theoretical basis for ensuring the health and safety of astronauts.
