# Erratum: A Current Overview of the Biological Effects of Combined Space Environmental Factors in Mammals

**DOI:** 10.3389/fcell.2022.943304

**Published:** 2022-06-02

**Authors:** Frontiers Production Office

**Affiliations:** Frontiers Media SA, Lausanne, Switzerland

**Keywords:** microgravity, space radiation, hypomagnetic field, space exploration, combined biological effect

Due to a production error, there was an error regarding the affiliation for Weiwei Pei. Their second affiliation was listed as “School of Radiation Medicine and Protection, Suzhou University Medical College of Soochow University, Suzhou, China”. The corrected affiliation is “School of Radiation Medicine and Protection, Suzhou Medical College of Soochow University, Suzhou, China”. The publisher apologizes for this mistake.

